# Integrative genomic analyses of a novel cytokine, interleukin-34 and its potential role in cancer prediction

**DOI:** 10.3892/ijmm.2014.2001

**Published:** 2014-11-12

**Authors:** BO WANG, WENMING XU, MIAOLIAN TAN, YAN XIAO, HAIWEI YANG, TIAN-SONG XIA

**Affiliations:** 1 Department of Medical Oncology, The Eastern Hospital of The First Affiliated Hospital, Sun Yat-Sen University, Guangzhou, Guangdong 510700, P.R. China; 2 Department of Endocrinology, The Eastern Hospital of The First Affiliated Hospital, Sun Yat-Sen University, Guangzhou, Guangdong 510700, P.R. China; 3 Department of Urology, The First Affiliated Hospital of Nanjing Medical University, Nanjing, Jiangsu 210029, P.R. China; 4 Department of Breast Surgery, The First Affiliated Hospital of Nanjing Medical University, Nanjing, Jiangsu 210029, P.R. China

**Keywords:** interleukin-34, comparative genomics, cancer, prognosis, meta-analysis

## Abstract

Interleukin-34 (IL-34) is a novel cytokine, which is composed of 222 amino acids and forms homodimers. It binds to the macrophage colony-stimulating factor (M-CSF) receptor and plays an important role in innate immunity and inflammatory processes. In the present study, we identified the completed IL-34 gene in 25 various mammalian genomes and found that IL-34 existed in all types of vertebrates, including fish, amphibians, birds and mammals. These species have a similar 7 exon/6 intron gene organization. The phylogenetic tree indicated that the IL-34 gene from the primate lineage, rodent lineage and teleost lineage form a species-specific cluster. It was found mammalian that IL-34 was under positive selection pressure with the identified positively selected site, 196Val. Fifty-five functionally relevant single nucleotide polymorphisms (SNPs), including 32 SNPs causing missense mutations, 3 exonic splicing enhancer SNPs and 20 SNPs causing nonsense mutations were identified from 2,141 available SNPs in the human IL-34 gene. IL-34 was expressed in various types of cancer, including blood, brain, breast, colorectal, eye, head and neck, lung, ovarian and skin cancer. A total of 5 out of 40 tests (1 blood cancer, 1 brain cancer, 1 colorectal cancer and 2 lung cancer) revealed an association between IL-34 gene expression and cancer prognosis. It was found that the association between the expression of IL-34 and cancer prognosis varied in different types of cancer, even in the same types of cancer from different databases. This suggests that the function of IL-34 in these tumors may be multidimensional. The upstream transcription factor 1 (USF1), regulatory factor X-1 (RFX1), the Sp1 transcription factor 1, POU class 3 homeobox 2 (POU3F2) and forkhead box L1 (FOXL1) regulatory transcription factor binding sites were identified in the IL-34 gene upstream (promoter) region, which may be involved in the effects of IL-34 in tumors.

## Introduction

Cytokines are glycosylated proteins that allow communication among various cell types involved in immune response. Interleukins (ILs) are cytokines mainly produced by T-cells, as well by monocytes, macrophages and endothelial cells (1,2). The different ILs share special biochemical or functional characteristics and are numbered in order of their identification. The emergence of new technologies is translating into a steady increase in the number of known molecules (3). In 2008, Lin *et al* (4) produced 3,400 recombinant secreted proteins that encode secreted proteins and extracellular domains of transmembrane proteins in 293T cells and examined their activities based on human monocyte screening assays. Subsequently, the authors (4) discovered a novel cytokine, IL-34. The human IL-34 protein is composed of 222 amino acids, has a molecular mass of 39 kDa and forms homodimers. It binds to the macrophage colony-stimulating factor (M-CSF) receptor, c-FMS (also known as CSF-1 receptor), expressed on the cell surface of human monocytes and has a stronger, although short-lived effect compared to M-CSF. IL-34 has been shown to be involved in the process of osteoclastogenesis and rheumatoid arthritis (RA) (5–8). IL-34 has been shown to promote the proliferation, survival and differentiation of monocytes and macrophages, the release of pro-inflammatory chemokines, and thereby plays an important role in innate immunity and inflammatory processes. It also plays an important role in the regulation of osteoclast proliferation and differentiation, and in the regulation of bone resorption (5–8).

IL-34 and M-CSF both signal via the same receptor, the M-CSF receptor. Although IL-34 and M-CSF show no appreciable similarity in their primary structure, they are evolutionally distant ligands, but are structurally related (9). There is evidence indicating that the M-CSF-IL-34-c-FMS axis is involved in the initiation, growth and metastasis of tumors (10,11). M-CSF levels may constitute a useful biomarker for a number of types of cancer, as it is expressed at high levels in a number of types of cancer, including breast cancer, ovarian cancer and colorectal carcinoma and its expression correlates with a poor prognosis (12). The direct inhibition of M-CSF or the inhibition of c-FMS kinase activity can lead to significant changes in the growth of grafted tumors (13.14). Tumor-associated macrophages are the most abundant component of the leukocyte infiltrate of solid tumors. In M-CSF-deficient mice (M-CSF^op/op^ or M-CSF^−/−^), the growth of the primary tumor and the metastatic spread of tumor cells has been shown to be significantly reduced due to the inability of angiogenesis to feed the tumors (12–15).

However, studies on the role of IL-34 in tumorigenesis. In the present study, we identified the IL-34 gene in various mammalian genomes by comparative genomic analyses. The conserved transcription factor-binding sites within the promoter region of the human IL-34 gene were then searched. Analyses of the expression data, functional relevant single nucleotide polymorphisms (SNPs) and comparative proteomic analysis were also conducted. Furthermore, a meta-analysis of the prognostic value of the IL-34 gene in various types of cancer was performed.

## Materials and methods

### Identification of the novel IL-34 gene in vertebrate genomes and integrative genomic analyses

All the IL-34 gene and amino acid sequences were obtained from the Ensembl database (http://www.ensembl.org/index.html), based on orthologous and paralogous relationships. The gained IL-34 sequences were applied as queries to search the IL-34 gene using BLAST at the National Center for Biotechnology Information (NCBI), in order to confirm whether their best hit was an IL-34 gene (16–18). The number and length of IL-34 exons and introns in all competent sequences were investigated for exon-intron conservation analyses. The number, length and structures of the exons and introns in IL-34 in all species were also collected from the Ensembl database (http://www.ensembl.org/index.html). Conserved transcription factor-binding sites within the promoter region of the human IL-34 gene were obtained from SABiosciences’ proprietary database which combines Text Mining Application and data from the UCSC Genome Browser (19–21).

### Comparative proteomic analysis of IL-34 protein

The protein coding sequences of IL-34 were aligned using ClustalW software implemented in MEGA 5.05. We constructed a maximum likelihood (ML) tree of IL-34 amino acid sequences using MEGA 5.05 with the optimal model (Kimura 2-parameter model). For the relative support of the internal node, bootstrap analysis was performed with 1,000 replications for ML reconstructions (22). The program CodeML implemented in the PAML 4.7 software package was used to investigate whether the IL-34 protein is under positive selection (23). The site-specific model was exerted using likelihood ratio tests (LRTs) to compare the M7 (null model) with the M8 model. M7 is a null model that does not allow for any codons with ω >1, whereas the M8 model allows for positively selective sites (ω >1). When the M8 model was fitted to the data more efficiently (P-value <0.05) than the null model (M7), the presence of sites with ω >1 was suggested. On the contrary, the results of P-value >0.05 proved the absence of sites with ω >1. Twice the log likelihood difference between the two compared models (2Δl) was compared against χ^2^ with critical values of 5.99 and 9.21 at 0.05 and 0.01 significance levels, respectively, as previously described (24).

### Functionally relevant SNP evaluation of the human IL-34 gene and identification of somatic mutations in human cancer

Functionally relevant SNPs of the human IL-34 gene were identified as previously described (16–21). The SNPs were extracted from the Ensembl (http://www.ensembl.org) and the NCBI SNPdb (http://www.ncbi.nlm.nih.gov) databases. The SNPs that disrupted exonic splicing enhancer/exonic splicing silencer (ESE/ESS) motifs and cause missence mutations were also identified. The identification of somatic mutations of the human IL-34 gene in human cancer was conducted in the Catalogue of Somatic Mutations in Cancer (COSMIC), a database for mining complete cancer genomes in the catalogue of somatic mutations in cancer (25).

### In silico expression analyses of the human IL-34 gene

Expressed sequence tags (ESTs) derived from the human IL-34 gene were searched for using the BLAST programs as previously described (26–29). The human IL-34 gene (NM_152456) was used as query sequences for the BLAST programs. The expression profiles for normal human tissues were obtained from GeneAnnot (30) and ArrayExpress (31) databases. Northern analysis of the NCBI uniGene dataset was also performed (19–21).

### Meta-analysis of the prognostic value of the IL-34 gene in cancer

A database termed ‘PrognoScan’ has been previously developed (32). This database includes a large collection of publicly available cancer microarray datasets with clinical annotation, as well as a tool for assessing the biological association between gene expression and prognosis. PrognoScan employs the minimum P-value approach for grouping patients for survival analysis. PrognoScan provides a powerful platform for evaluating potential tumor markers and therapeutic targets and is publicly accessible at http://www.sabiosciences.com. The human IL-34 gene was used as an input source as a query and the data were collected for analysis.

## Results

### Comparative proteomic analysis of IL-34 protein identified in vertebrate genomes

**Figure 1:**
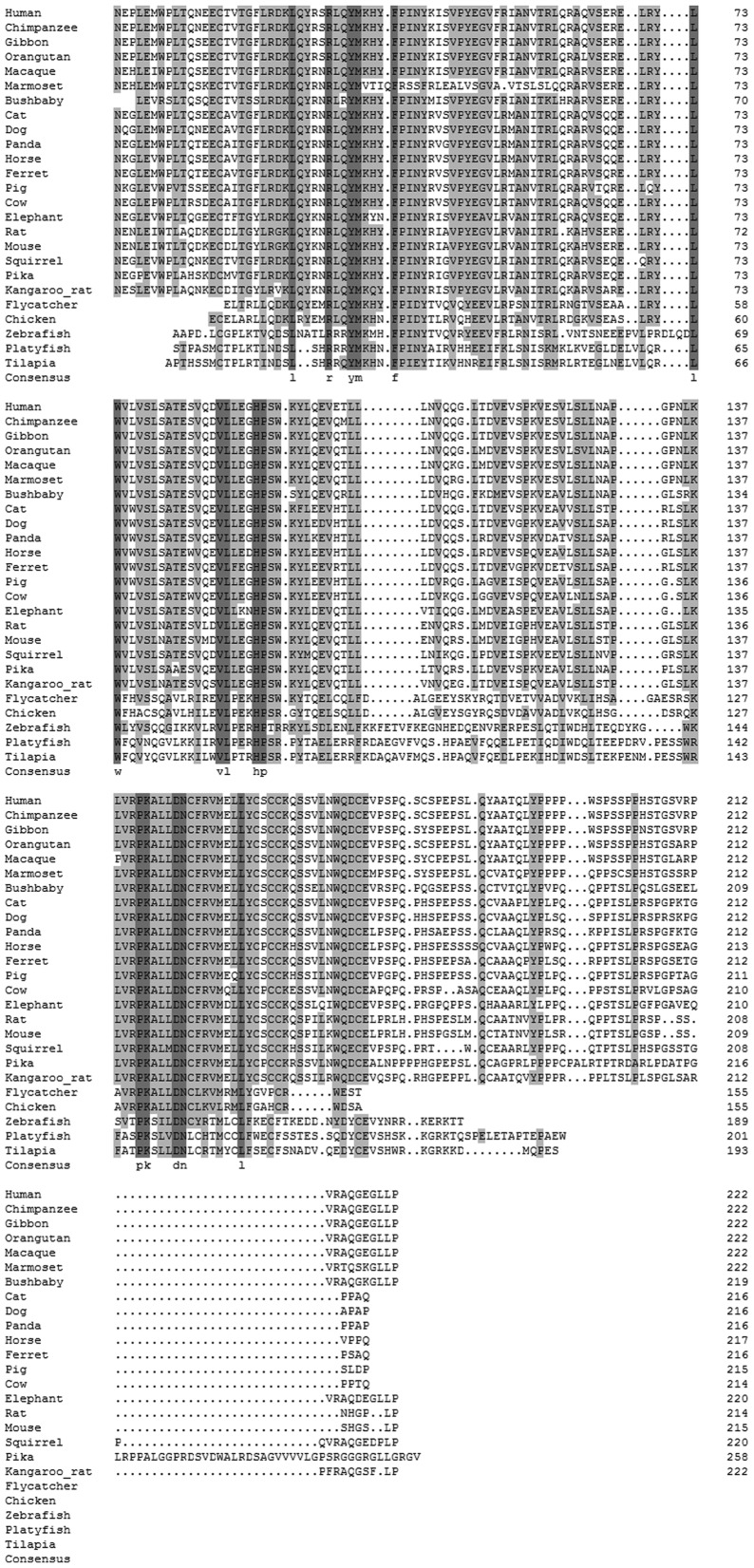
Sequence and structural alignment of vertebrate interleukin-34 (IL-34). All the IL-34 gene and protein sequences were collected from the Ensembl database and confirmed by BLAST at the National Center for Biotechnology Information (NCBI). The complete IL-34 gene was identified in 25 various mammalian genomes, such as the human, chimpanzee, gibbon, macaque, orangutan, marmoset, bushbaby, pika, squirrel, rat, mouse, kangaroo rat, elephant, cat, dog, panda, ferret, pig, horse, cow, flycatcher, chicken, zebrafish, platyfish and tilapia genomes.

**Figure 2:**
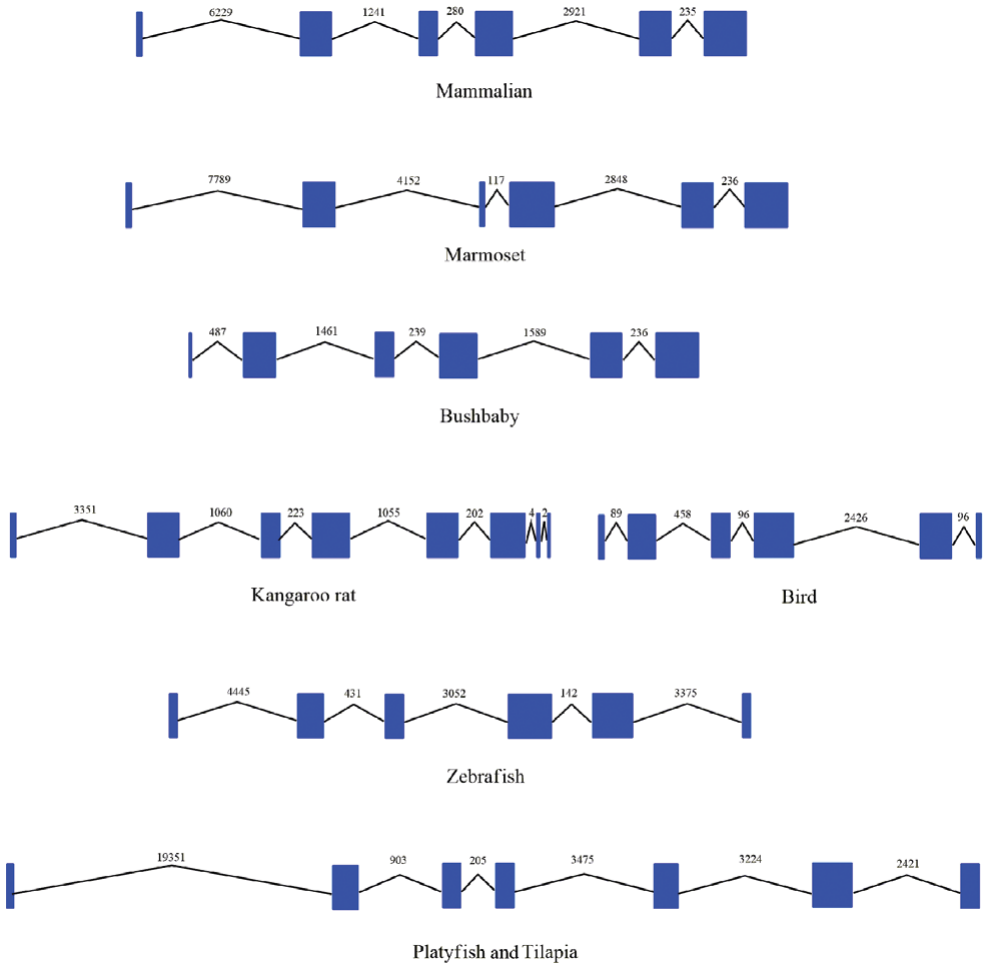
The phylogenetic tree was constructed according to the protein coding sequences of interleukin-34 (IL-34) using the maximum likelihood method. The IL-34 gene from the primate lineage, rodent lineage and teleost lineage formed a species-specific cluster.

**Figure 3:**
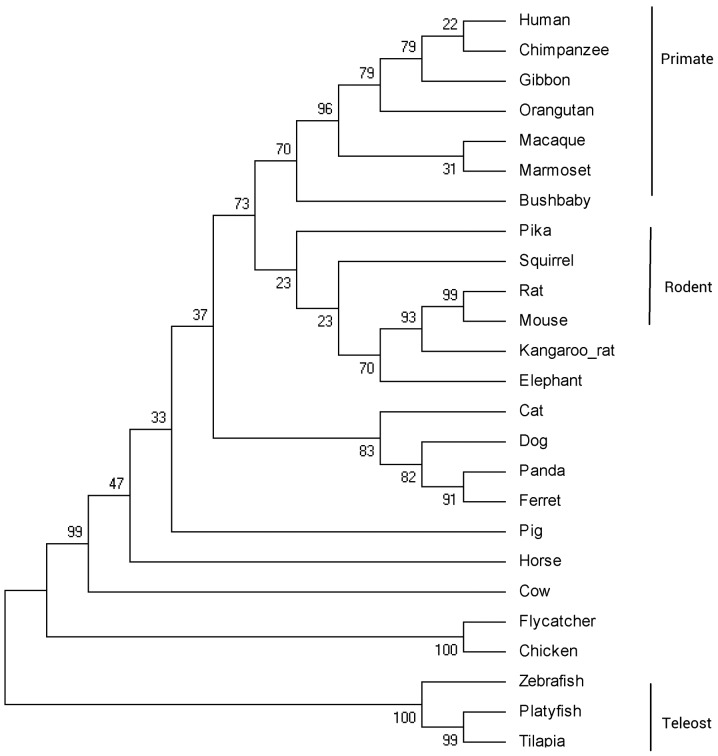
Exon-intron conservation of the interleukin-34 (IL-34) gene among different species. In the majority of vertebrates, the IL-34 gene showed an exon-intron conservation with 5 introns and similar sizes of each intron. With exception, there are 8 exons and 7 introns in the IL-34 gene in the kangaroo rat. Moreover, in platyfish and tilapia, the IL-34 gene contains 7 exons and 6 introns.

All the IL-34 gene and protein sequences were collected from the Ensembl database and confirmed by BLAST at NCBI. The complete IL-34 gene was identified in the human, chimpanzee, gibbon, macaque, orangutan, marmoset, bushbaby, pika, squirrel, rat, mouse, kangaroo rat, elephant, cat, dog, panda, ferret, pig, horse, cow, flycatcher, chicken, zebrafish, platyfish and tilapia. The sequence and structural alignment of IL-34 is illustrated in Fig. 1. The phylogenetic tree was constructed according to the protein coding sequences of IL-34 using the ML method (Fig. 2). The IL-34 gene from the primate lineage, rodent lineage and teleost lineage forms a species-specific cluster. The exon-intron information collected from the Ensembl database is presented in Table I and Fig. 3. In the majority of genomes, the IL-34 gene has 6 exons with similar lengths in different species (Table I). In the majority of vertebrates, the IL-34 gene shows exon-intron conservation with 5 introns and similar sizes of each intron. With exception, there are 8 exons and 7 introns in the IL-34 gene in the kangaroo rat. Moreover, the IL-34 gene in the platyfish and tilapia contains 7 exons and 6 introns. Thus, the intron deletions of the IL-34 gene may occur during the evolutionary process in fish. Furthermore, site-specific tests for positive selection were performed for vertebrate, mammalian, primate and mammalian excluding primate, rodent and teleost lineages. Although some positive selection sites were computed, only the 2Δl of M7 and M8 of mammalian IL-34 was >5.99, indicating that the M8 model was more efficient than the M7 model in fitting the data. It seemed that mammalian IL-34 was under positive selecetion pressure with the identified positively selected site, 196Val (Table II).

### Expression profile of the human IL-34 gene

By EST sequence searching, the human IL-34 gene was found to be expressed in the adult and fetal brain, the hippocampus, spleen, embryonic stem cells, heart, medulla, lung, testes, ovaries, metastatic chondrosarcoma, epidermis, keratinocytes, osteoarthritic cartilage, adipose tissue, choroid, eyes, amygdala, kidneys, thymus, small intestine, hypothalamus, islets of Langerhans, glioblastoma and the retinal pigment epithelium. The investigation of available microarray analyses and ‘virtual northern blot analysis’ revealed a predominant expression of IL-34 in the lymph nodes, brain, heart, skeletal muscle, colon, adipocyte, kidneys, liver, lungs, thyroid, adrenal gland, ovaries, prostate and testes. When performing a search in the PrognoScan database, the human IL-34 gene was also found to be expressed in various types of cancer, such as blood, brain, breast, colorectal, eye, head and neck, lung, ovarian and skin cancer.

### Comparative genomic anlaysis of the human IL-34 gene

The upstream transcription factor 1 (USF1), regulatory factor X-1 (RFX1), the Sp1 transcription factor 1, POU class 3 homeobox 2 (POU3F2) and the forkhead box L1 (FOXL1) regulatory transcription factor binding sites were identified in the IL-34 gene upstream (promoter) region.

### Functionally relevant SNP evaluation of the human IL-34 gene and identification of somatic mutations in human cancer

A total of 2,141 available SNPs were identified in the human IL-34 gene. Among these SNPs, a total of 55 SNPs were functionally relevant; these included 32 SNPs causing missense mutations, 3 exonic splicing enhancer SNPs and 20 SNPs causing nonsense mutations (Table III). As presented in Table IV, by performing a search of the COSMIC database, we identified 18 somatic mutations of the IL-34 gene in cancer.

### Meta-analysis of the prognostic value of the human IL-34 gene in cancer

When the name of a gene is submitted, PrognoScan displays a summary in table format of tests for the gene with columns for dataset, cancer type, subtype, endpoint, cohort, contributor, array type, probe ID, number of patients, optimal cutpoint, Pmin and Pcor. Among the databases which detected the expression of the IL-34 gene, 5 out of 40 tests revealed an association between the expression of the IL-34 gene and cancer prognosis (blood cancer, 1/4; brain cancer, 1/4; breast cancer, 0/11; colorectal cancer, 1/7; eye cancer, 0/1; head and neck cancer, 0/3; lung cancer, 2/6; ovarian cancer, 0/3; and skin cancer, 0/1) with a 5% significance level (Table V). Among the two types of lung cancer, the lower expression of the IL-34 gene was related to poor survival and was found in non-small cell lung cancer (NSCLC) case (GSE8894). However, a higher expression of the IL-34 gene was related to poor survival in a case of adenocarcinoma (GSE31210). As for blood cancer cases and colorectal cancer, we found that a lower expression of the IL-34 gene was associated with poor survival. However, in the brain cancer cases, a higher expression of the IL-34 gene was related to poor survival.

## Discussion

IL-34 was identified by functional screening of a library of secreted proteins, based on its ability to support human monocyte survival and to promote, with the same efficiency as M-CSF, the formation of the colony forming unit-macrophage (CFU-M) in human bone marrow cell cultures (4).

In the present study, we identified the complete IL-34 gene in 25 various mammalian genomes, including the human, chimpanzee, gibbon, macaque, orangutan, marmoset, bushbaby, pika, squirrel, rat, mouse, kangaroo rat, elephant, cat, dog, panda, ferret, pig, horse, cow, flycatcher, chicken, zebrafish, platyfish and tilapia genomes. In addition, we found that IL-34 existed in all types of vertebrates, including fish, amphibians, birds and mammals. The IL-34 gene has a similar 7 exon/6 intron gene organization in various species, and genes in the IL-34 loci were syntenically conserved (33,34). The phylogenetic tree demonstrated that the IL-34 gene from the primate lineage, rodent lineage and teleost lineage formed a species-specific cluster. From the alignment and phylogenetic tree, mammalian IL-34 was conversed among vertebrate genomes, suggesting that the function of the IL-34 gene plays an important physiological role in all vertebrates in the long evolutionary process. It seemed that the mammalian IL-34 gene was under positive selection pressure with the identified positively selected site, 196Val. This is in accordance the with multiple biological functions of a cytokine, which plays a key role in the immune system.

IL-34 mRNA is widely expressed in various types of tissue, including tissue of the heart, brain, lung, liver, kidneys, thymus and spleen (4). Accordingly, by EST sequence searching, the IL-34 gene was also found to be expressed in various other types of tissues and cells, including the hippocampus, embryonic stem cells, medulla, testes, ovaries, metastatic chondrosarcoma, epidermis, keratinocytes, osteoarthritic cartilage, adipose tissue, choroid, eyes, amygdala thymus, small intestine, hypothalamus, islets of Langerhans, glioblastoma and the retinal pigment epithelium. This suggests that the IL-34 gene is widely expressed in many types of tissues and organs. The investigation of available microarray analyses and ‘virtual northern blot analysis’ confirmed the predominant expression of IL-34 in the lymph nodes, brain, heart, skeletal muscle, colon, adipocyte, kidneys, liver, lung, thyroid, adrenal gland, ovaries, prostate and testes. A total of 55 functionally relevant SNPs, including 32 SNPs causing missense mutations, 3 exonic splicing enhancer SNPs and 20 SNPs causing nonsense mutations were identified from 2,141 available SNPs in the human IL-34 gene, which may affect the multiple functions of IL-34. However, the effects of these SNPs on the physiological and pathological function of IL-34 require further investigation.

IL-34 and M-CSF both signal via the same receptor, the M-CSF receptor, c-FMS. It has been shown that M-CSF is expressed at high levels in many types of tumor, including breast cancer, ovarian cancer and colorectal carcinoma and correlates with a poor prognosis (10–15). However, studies on the role of IL-34 in tumor development are limited. In the present study, we firstly found that IL-34 was indeed expressed in various types of cancer, such as blood, brain, breast, colorectal, eye, head and neck, lung, ovarian and skin cancer. A total of 5 out of 40 tests (1 blood cancer, 1 brain cancer, 1 colorectal cancer and 2 lung cancer) revealed an association between IL-34 gene expression and cancer prognosis. The mechanisms responsible for the involvement of IL-34 in the progression of these tumors require further investigation. It should be noted that the association between the expression of IL-34 and prognosis varies in different types of cancer, even in the same type of cancer from different databases. This suggests that the function of IL-34 in these tumors may be multidimensional, not only functioning as a tumor inhibitor or promoter. Moreover, we identified 18 somatic mutations of IL-34 in cancer tissue in the present study. The mechanisms through which these mutations affect tumor formation require further investigation. These data suggest that IL-34, similar to M-CSF, is involved in tumor formation.

USF1, RFX1, Sp1, POU3F2 and FOXL1 regulatory transcription factor binding sites were identified in the IL-34 gene upstream (promoter) region. USF-1 is an important transcription factor that participates in glucose metabolism and tumorigenesis. It has a negative effect on cell proliferation in some cell types and stabilizes the p53 protein and promotes a transient cell cycle arrest, in the presence of DNA damage (34,35). RFX1 is unique transcription factor that contains a highly conserved 76-amino-acid DNA binding domain. RFX1 can directly regulate CD44 expression (36,37). This mechanism may contribute to the effects of RFX1 on the proliferation, survival and invasion of glioblastoma cells. Sp1 is a member of the Sp/Krüppel-like factor (KLF) family of transcription factors that play a critical role in embryonic and early postnatal development, differentiation, cell cycle regulation and in multiple diseases, including cancer (38–41). POU domain transcription factors are present in a number of cell lineages where they perform various functions, either as ubiquitous regulators of ‘housekeeping’ genes, or as developmental- and lineage-specific coordinators of cell fate decisions (42). POU3F2 has been shown to be responsive to MAPK pathway activation and to modulate the levels of microphthalmia-associated transcription factor (MITF) so as to suppress the differentiated melanocytic phenotype and to enhance tumor metastasis (29). FOXL1 is located at the junction of multiple signaling pathways and plays critical roles in a variety of physiological and pathological processes, including cancer development. These tumor-related transcriptional factors may be involved in the effects of IL-34 in tumors (28,43,44).

## Figures and Tables

**Table I:** Exon and intron lengths of IL-34.

	Length (bp)															
	
Species	Exon 1	Intron 1	Exon 2	Intron 2	Exon 3	Intron 3	Exon 4	Intron 4	Exon 5	Intron 5	Exon 6	Intron 6	Exon 7	Intron 7	Exon 8	Total exons
Human	28	7,562	134	1,930	78	280	162	2,496	136	243	191	-	-	-	-	729
Chimpanzee	28	8,179	134	1,926	78	280	162	2,495	136	250	191	-	-	-	-	729
Gibbon	28	6,472	134	1,930	78	280	162	2,538	136	250	191	-	-	-	-	729
Orangutan	28	7,643	134	2,042	78	280	162	2,563	136	258	191	-	-	-	-	729
Macaque	28	7,369	134	2,064	78	288	162	2,531	136	239	191	-	-	-	-	729
Marmoset	28	7,789	142	4,152	27	117	205	2,848	136	236	191	-	-	-	-	729
Bushbaby	15	487	147	1,461	78	239	162	1,589	136	236	170	-	-	-	-	711
Cat	28	6,405	134	787	78	285	162	2,902	136	230	173	-	-	-	-	711
Dog	28	6,254	134	940	78	281	162	2,366	136	238	173	-	-	-	-	711
Panda	28	5,018	134	797	78	284	162	2,066	136	230	173	-	-	-	-	711
Horse	28	5,227	134	841	78	284	162	2,162	136	217	176	-	-	-	-	714
Ferret	28	4,504	134	771	78	306	162	2,043	136	234	173	-	-	-	-	711
Cow	28	5,849	134	751	78	261	162	2,879	133	229	170	-	-	-	-	705
Rat	28	5,484	134	1,125	78	269	159	5,154	136	264	170	-	-	-	-	705
Mouse	28	5,672	134	684	78	258	162	5,463	136	195	170	-	-	-	-	708
Squirrel	28	5,565	134	789	78	277	162	3,231	136	210	185	-	-	-	-	723
Kangaroo rat	28	3,351	134	1,060	78	223	162	1,055	136	202	160	4	16	2	15	729
Flycatcher	28	92	104	293	78	84	174	3,627	139	99	20	-	-	-	-	543
Chicken	28	86	104	623	78	108	174	1,225	133	82	20	-	-	-	-	537
Zebrafish	40	4,445	107	431	81	3,052	201	142	169	3,375	44	-	-	-	-	642
Platyfish	40	26,738	104	1,344	78	256	78	4,479	108	2,376	184	4,042	86	-	-	678
Tilapia	37	11,964	104	461	78	154	78	2,170	108	4,072	184	799	59	-	-	648

IL-34, interleukin-34.

**Table II:** Site-specific tests for positive selection on IL-34.

Species	Models	Estimates of parameters	lnL	2Δl	Positively selected sites
Vertebrate	M7	P=1.03903, Q=5.39585	−4659.912970	2.057682	NS
	M8	P0=0.98039, P=1.20110, Q=7.10944 (P1=0.01961) w=1.00000	−4657.855288		
Mammalian	M7	P=0.48941, Q=1.45186	−4490.139825	6.824409	196 V^a^
	M8	P0=0.96625, P=0.63368, Q=2.34086 (P1=0.03375), w=1.81330	−4483.315416		
Primate	M7	P=0.01895, Q=0.02238	−1715.925824	0.033788	NS
	M8	P0=0.56073, P=0.00997, Q=0.16501 (P1=0.43927) w=1.00000	−1715.959612		
Mammalian excluding primate	M7	P=0.30844, Q=1.06769	−3530.180011	2.160234	200 Q^a^
	M8	P0=0.98088, P=0.35332, Q=1.40393 (P1=0.01912) w=2.08098	−3528.019777		
Rodent	M7	P=0.33256, Q=1.26857	−1743.972997	0.000104	NS
	M8	P0=0.99999, P=0.33225, Q=1.26708 (P1=0.00001) w=3.17132	−1743.973101		
Teleost	M7	P=0.55893, Q=1.64564	−1656.009963	0.088367	NS
	M8	P0=0.99243, P=0.58684, Q=1.81573 (P1=0.00757) w=8.77551	−1655.921596		

a The positively selected sites were identified with posterior probability ≥0.95 using the Bayes empirical Bayes (BEB) approach.

lnL, the log-likelihood difference between the two models; 2Δl, twice the log-likelihood difference between the two models (in all species, 2Δl <9.21, the P-value is more than the significance level 0.05, indicating that the M8 model was more efficient than the M7 model); NA, not allowed; NS, not shown (sites under positive selection did not reach the significance level of 0.95). IL-34, interleukin-34.

**Table III:** Functionally relevant SNP evaluation of the human IL-34 gene.

SNP ID	Chr 16 position sequence	Sequence	Type	Amino acid change
rs200158701	70680854(+)	CCATGC/TCCCGG	mis	PS
rs192337001	70680866(+)	GCTTCA/CCCTGG	mis	TP
rs139133476	70688459(+)	CCTTGG/CCGTGG	mis	AG
rs142890682	70688461(+)	TTGGCG/ATGGCC	mis	MV
rs118062333	70690511(+)	AACACT/CACTTC	mis	HY
rs200597979	70690960(+)	GGGCCA/GCCCAT	mis	HR
rs8046424	70690989(+)	AGGTGC/GAGACG	mis	QE
rs187166563	70693576(+)	CCCAGA/GGCCAA	mis	EG
rs142214904	70693626(+)	GCTTCC/TGGGTC	mis	RW
rs7206509	70693945(+)	GCCAAG/CTCCTC	mis	TS
rs201277640	70693984(+)	GTATGC/TGGCCA	mis	AV
rs202122982	70694001(+)	TGTACC/TCTCCG	mis	PS
rs148286339	70694011(+)	GCCCCC/TGTGGT	mis	PL
rs141513638	70694056(+)	GAGGCC/TGGTCA	mis	PL
rs112639369	70694073(+)	AGGGCG/AAGGGC	mis	KE
rs1444643201	70694076(+)	GCGAGG/AGCCTC	mis	SG
rs367851338	70693627(+)	CTTCCA/GGGTCA	mis	QR
rs368143418	70690933(+)	TGAGTC/TGGTGC	mis	SL
rs374665339	70690963(+)	CCACCC/TATCCT	mis	PL
rs368923655	70691023(+)	CCTCAC/TGGTGA	mis	TM
rs368367274	70693597(+)	GGTGCA/GGCCCA	mis	QR
rs200891924	70693560(+)	TGTCCC/ATCTTG	mis	IL
rs372998917	70694041(+)	CTCCAC/TGGGCT	mis	TM
rs370436386	70690927(+)	TGCCAC/TTGAGT	mis	TL
rs144427482	70690571(+)	CCAACG/ATCACC	mis	IV
rs201108464	70693569(+)	TGAATG/ACCCCA	mis	TA
rs144144426	70690541(+)	GTGTGC/TCTTAC	mis	PS
rs377411431	70690885(+)	CGAGCG/TGGAGC	mis	RL
rs369011177	70680875(+)	GGCTGC/TGCTGT	mis	RC
rs145782768	70693979(+)	TTGCAG/CTATGC	mis	HQ
rs201784459	70694005(+)	CCCTCC/TGCCCC	mis	PL
rs200488835	70693655(+)	TCCTGC/GTGTAA	mis	CW
rs3813904	70680744(+)	TGACTG/CAGTGA	ese	
rs3813905	70680850(+)	ACCACC/GATGCC	ese	
rs4985556	70694000(+)	CTGTAC/ACCTCC	ese	

A total of 55 SNPs were functionally relevant; including 32 SNPs causing missense mutations, 3 exonic splicing enhancer SNPs and 20 SNPs causing nonsense mutations. SNP, single nucleotide polymorphism; IL-34, interleukin-34; mis, missense; ese, exonic splicing enhancer.

**Table IV:** Somatic mutations of IL-34 in cancer tissue.

Position (AA)	Mutation (CDS)	Mutation (amino acid)	Mutation ID (COSM)	Count	Mutation type
4	c.11G>A	p.G4D	COSM973055	1	Substitution - missense
9	c.25C>T	p.R9C	COSM3691133	1	Substitution - missense
33	c.99G>A	p.E33E	COSM704311	1	Substitution - coding silent
38	c.114G>A	p.T38T	COSM973057	1	Substitution - coding silent
42	c.125_126GG>AA	p.R42Q	COSM143555	1	Substitution - missense
59	c.176C>T	p.P59L	COSM108032	1	Substitution - missense
61	c.182A>G	p.N61S	COSM3387573	1	Substitution - missense
104	c.311C>T	p.S104L	COSM435667	1	Substitution - missense
155	c.465C>T	p.N155N	COSM328594	9	Substitution - coding silent
170	c.508C>T	p.R170W	COSM194870	1	Substitution - missense
183	c.549C>A	p.S183R	COSM3402448	1	Substitution - missense
197	c.589C>T	p.Q197*	COSM1379321	1	Substitution - nonsense
197	c.590A>G	p.Q197R	COSM1379322	1	Substitution - missense
197	c.591G>A	p.Q197Q	COSM40324	1	Substitution - coding silent
208	c.623C>T	p.A208V	COSM417292	2	Substitution - missense
208	c.624G>A	p.A208A	COSM1177412	1	Substitution - coding silent
217	c.651G>A	p.P217P	COSM1379323	1	Substitution - coding silent
229	c.686C>T	p.S229L	COSM417291	3	Substitution - missense

IL-34, interleukin-34.

**Table V:** Dataset content from the PrognoScan database demonstrating an association between microarray analyses in IL-34 and cancer prognosis.

Database	Case type	Subsyte	No. of patients	Endpoint	Cutpoint	P-value	Prognosis	(Refs.)
GSE12417-GPL570	Blood cancer	AML	79	Overall survival	0.18	0.028	1	(45)
GSE4412-GPL97	Brain cancer	Glioma	74	Overall survival	0.72	0.003	2	(46)
GSE17537	Colorectal cancer		55	Overall survival	0.38	0.04	1	(47)
GSE31210	Lung cancer	Adenocarcinoma	204	Relapse-free survival	0.89	0.03	2	(48)
GSE8894	Lung cancer	NSCLC	138	Relapse-free survival	0.4	0.0002	1	(49)

A total of 5 out of 40 tests showed an association between the expression of the IL-34 gene in microarray analysis and cancer prognosis (blood cancer, 1/4; brain cancer, 1/4; breast cancer, 0/11; colorectal cancer, 1/7; eye cancer, 0/1; head and neck cancer, 0/3; lung cancer, 2/6; ovarian cancer, 0/3; and skin cancer, 0/1) with a 5% significance level. IL-34, interleukin-34; NSCLC, non-small cell lung cancer.
